# Dr. Gardette on the Propriety of Abolishing Dental Colleges, and Substituting for Them a Lectureship on Dentistry in Each of the Medical Schools

**Published:** 1851-04

**Authors:** 


### EDITORIAL DEPARTMENT.

Dr. Gardette *on the Propriety of Abolishing Dental Colleges, and substituting for them a Lectureship on Dentistry in each of the Medical Schools.*

We take the following article entire, from the April number for the present year, of the American Journal of Medical Sciences:

"*On the Importance of Establishing a Lectureship on Dental Surgery in Medical Colleges.* By E. B. Gardette.

"The writer, a practicing dentist of Philadelphia, begs leave respectfully to call the attention of the trustees and medical faculties of the medical schools of the United States to the propriety and advantages of establishing an adjunct professorship to the chair of surgery, in which the specialty of dental surgery may be taught to the medical student seeking knowledge in your institutions.

"In making this suggestion, he indulges the confident belief that the existence of such a chair would be no less useful to those who may be compelled to practice some branch of dental surgery, as part of their duties in general surgery, than to the smaller number who may determine to embrace that specialty as their profession.

"The writer would offer for your consideration some reflections that seem to render this proposal consistent, not only with the wants of the student of medicine and the public, but with the duty and the interests of the medical schools which may act upon this request in such form as seems most proper to their own judgment.

"It will scarcely be assumed by any trustee, and still less by any member of a medical faculty, that the profession of the dentist or its duties, are less important to mankind than any other of the specialties of surgery—whether the oculist, the aurist, or the lithotomist; and therefore it is needless to consider such a question open for debate, or requiring argument in a country, especially, where the services of good dentists have been so widely and universally needed. It being granted, then, that the science itself is of equal importance with the other departments of surgery, it is an undeniable truth that, whilst all other specialties are taught and included in the professorships of medical schools generally, the principles and practice of dental surgery have received no attention, or certainly none that would permit the graduate of medicine to feel that he was qualified for the simplest duty of the dentist.

"Such a state of things was perhaps an unavoidable evil when men could not be found who were competent to teach the principles of our science, and when there was need of a comparatively small number of dentists; and hence no such aid was then called for from the medical colleges: but at this time there would seem to be required about twenty-five hundred dentists, more or less, throughout the United States, which is sufficiently proved by the fact that about that number are believed to be engaged in the practice of the profession, after some fashion or other—good, bad, and indifferent; and to supply an increasing demand for well-educated dental surgeons, as well as to raise the standard of that profession would appear to be a matter of common interest.

"It would seem an act of inexplicable unkindness to have thus singled out the profession of dentist from among the specialties of surgery, and forced it into an attitude of independent self-protection; but a gentleman who fulfils the duties of both medical practitioner and dentist with such distinguished abilities as to be an honor to both professions, has traced truthfully the origin of the neglect of which the dentist complains. The undersigned quotes from a valedictory address to the graduating class of Baltimore College of Dental Surgery, March, 1850, when speaking of the early periods of the existence of a medical faculty:—

"'They condemned, without stint, a calling they knew not how to practice, and a practice they knew not how to improve. Such of the faculty as were learned in their profession were found always competent and fully prepared to be oculists, aurists, or lithotomists, or to devote themselves to any other branch of the profession which their interest, inclination, or talents might determine, *except* that of dental surgery. This branch seemed to require something more than medical knowledge; it required great mechanical skill—an education of the hand as well as of the head—a kind of education they had not received and knew not where to acquire, and yet affected to despise. The necessities of the community cried aloud to them for help—a help which they could not bestow. This drove many sufferers to seek dental aid out of the medical profession, and to obtain that help which mechanical genius alone could supply. At this the profession seemed mortified and chagrined, and loudly mocked at those who dared to supply their delinquencies, and united as one man in deriding the uneducated dental mechanic. They first created the necessity for an empiric, and then croaked forth their withering contempt on the creature their own ignorance had made.'

"Thus has dental surgery been left to struggle through endless impediments and difficulties, instead of being regarded as a link, however humble, in the great chain of medical science, which should with patriarchal strength and harmony foster and embrace every branch of the healing art.

"It is now believed that, among the number of medical students who attend each of the schools, there are many who would gladly devote themselves to the specialty of dental surgery, and it is from among these, and at the early age at which they generally devote themselves to medical studies, that good dentists could be formed; they should still be required to earn and receive a diploma with the title of M. D., as a guarantee of fitness to practice and a claim to confidence as dentist. Even among the number that annually graduate, either to practice medicine generally, or limiting their attendance to their own families and dependents at the South, a correct knowledge of the theory and practice of dental surgery, such as might be derived from a course of lectures, would be highly valuable. And it is believed there would not only be a direct increase of good dentists, but a desirable addition to the number of those who should be competent to determine what constitutes a good dentist.

"The establishment of separate schools for each specialty in medical science (and why should there not be for the oculist, or the aurist, as well the dentist?) would appear to be the dismembering of a great family, thereby lessening its influence for good, as well as that power which is justly derived from an *esprit de corps* subsisting in every branch of an elevated and honorable profession. In this expression of opinion as to the ultimate tendency of dental colleges, the undersigned is actuated by no unkind or invidious feelings, but, on the contrary, he gives them due credit for the efforts they have made and are making to improve the profession; and to the Baltimore College of Dental Surgery he is personally grateful for acts of courtesy and kindness towards himself, and to the memory of his father.

"The educated dentists in the various parts of the United States are abundantly numerous, it is believed, to fulfil such duties as might be assigned them in each of our medical colleges. The students who now attend the dental schools would, in this event, be added to the number of matriculants in the medical classes of the country, and the need of separate institutions would cease.

"Frequent occasion for consultations between the physician, the surgeon and the dentist, has been felt by almost every practitioner of medicine, as a necessary consequence of the connection and sympathy reciprocally existing between the teeth and many serious disturbances of the general health: such sympathies would naturally seem to suggest the mutual dependence between the physician, the surgeon and the dentist, and should be a good reason for some similarity and sympathy in their modes of education. The benefits from such a state of things as the mind can readily anticipate would not rest here; medical men would sometimes, no doubt, receive important aid from the dentist, in tracing to their true origin many diseases of the head and face that now baffle their skill.

"Whatever action your medical faculty or board of trustees may see fit to take in reference to these suggestions, and the object they are designed to promote, the undersigned may at least respectfully urge that they would seem to demand fair and kindly consideration, as involving matters of deep concern to the community, for whose safety and advantage all medical learning is sought, and medical colleges instituted and fostered."

For the writer of the foregoing, we entertain great personal regard, and we regret, extremely, that our sense of duty compels us to controvert his opinions, and protest against the adoption of his suggestions. If Dr. Gardette were an ordinary man, wielding no greater influence than is commonly exerted by an individual member of our profession, we might have yielded to our personal feelings, and permitted his overture to the medical schools to pass without remonstrance. But such is not the case. The author of this, as it seems to us, suicidal proposition, is a gentleman who deservedly ranks high among us. His professional and social position give him uncommon importance; and we are not at liberty to estimate the danger of his proceeding at its intrinsic worth. There is reason to fear, moreover, that our silence might be construed into assent in quarters where we are anxious that no such mistake shall be made.

Dr. Gardette has, by this bold and honest expression of his opinion, compelled us to declare ours, and he will not complain that we do so as boldly and frankly as he has done.

This proposition is made at a most unfortunate time. After many years of vain endeavors on the part of a few enlightened dentists to improve the professional and social position of their particular specialty, by writings and private instructions, and attendance upon medical lectures, dental surgery was found to have settled down to a point of degradation, from which its extrication seemed hopeless. The better class of operators shunned fellowship with the rest, and in a state of sullen and fretful isolation, grieved over evils they knew not how to avert.

At this period, when all was dark and hopeless, the idea was conceived of establishing a Dental College, and professionalising dental surgery. The enterprise was undertaken by a few men, without the promise of patronage, or even the prospect of sympathy. When they began to lecture, they had two pupils; in four years they had less than a dozen—while dentists throughout the country were annually sending out hundreds of students from their offices. The truth must be told. But few, very few, of the educated class of dentists encouraged the undertaking, while very many derided it, and used all their influence to keep students away.

In the *medical profession*, from the first, the College found sympathy and active kindness, and it is only just to say, that for years the infant institution looked to physicians for aid to sustain them against the petty jealousy and covetousness of those who should have been its natural supporters.

After twelve years of endurance and toil, the institution began to show that it had within itself the elements of prosperity. A hundred graduated pupils had carried its name over the country, and demonstrated, by their skill and success, the advantages to be procured by attendance upon its instructions. The medical faculty had recognized it as a medical school, and welcomed its delegates to seats in their great annual convocation. The public congregated at its commencements, and pupils increased in its halls.

The beginning had been made. A profession of dental surgery, standing upon its own merits, respected for its own sake, had been recognized in the country. Opposition was beginning to grow weaker, and sympathy stronger, and the friends of the profession began to feel that the way up from its nadir had been opened, and that a few years would bring about a general ascent from degradation.

Just at this moment Dr. Gardette proposes to undo this work, to abandon all hope of making dental surgery anything, and to append what we can of it to medical colleges, where it would only serve as a mere whistle to allure a few more students to their halls.

The reasons which are given for this strange and startling proposition, are few and feeble; indeed, we can hardly perceive that they amount to reasons at all. Certainly Dr. Gardette should have shown that dental surgery could be taught by an adjunct professor equally well as by the five instructors who are now found necessary in a dental college. He should have shown that the likelihood of making good dentists would have been as great under the new method as the old. The kind and quality of instruction to be given should have formed at least an element in his scheme and an argument for his appeal. But of all this nothing is said. The only reason why Dr. Gardette wishes the dental profession to be abandoned, and a kind of dentalized medicine substituted, seems to be that he supposes there is degradation in the separation. In other words, he supposes that if dentists were graduates in medicine, dentists would be more respectable. Whether this would be so, is not left to conjecture. There are in the country many medical men who are practicing dentistry. Are they more respectable than others of equal education and skill? Are there not among them many whose operations are so far below mediocrity that Dr. Gardette would scorn to call them brethren?

As to the great advantage which would be conferred upon the public by teaching medical students a little collateral dentistry, we have our doubts. A little knowledge is a dangerous thing, especially a little knowledge in wielding such formidable engines as dental instruments.

When the first dental college was about going into operation, we were waited on by three members of the faculty of one of our oldest and most respectable medical schools, and tendered such a professorship as is suggested by Dr. Gardette, but we declined it upon the ground that no purely medical institution could afford the necessary facilities for thorough practical instruction in operative and mechanical dentistry, and that, consequently, if we accepted it, we would be employed in making the worst kind of quacks—men without skill, but with acknowledged pretensions to it.

It is useless, however, to multiply words about this matter. Practically, the scheme is *impossible*, and so it will be found whenever serious arrangements begin to be made to carry it into effect.

According to Dr. Gardette's scheme, all the medical students must be thoroughly instructed in dentistry. In the first place, they will not submit to any such thing; in the next place, no adjunct professor could so instruct them, if they would. The course of medical studies in the colleges is now so extended, that with all possible diligence, students cannot find time to get through with it in the four months session, and general clamor is demanding an extension of the time. How will they find time for theoretical, and mechanical, and operative dentistry? Again, dental students are to be thoroughly instructed in all the branches of medicine. Will they consent to devote sufficient time to obstetrics and *all* the other departments of medicine to become proficient in them, knowing that they will never turn much of this information to practical use, and, consequently, will forget faster than they acquire it.

Dr. Gardette does not propose that the term of attendance upon lectures shall be increased; but he expects that an adjunct professor will be able to instruct a class of students, numbering, perhaps, five hundred, in theoretical, mechanical and operative dentistry, by devoting, we suppose—and certainly no more can be granted—an hour a day to the purpose. In four months he would lecture about a hundred times; or during a hundred hours, which being divided into working days, would give ten days entire to dentistry; in which time, all necessary information is to be imparted—

all necessary skill acquired. If we suppose the student to attend two sessions we find his dental education completed in twenty days.

Now, in the Baltimore College of Dental Surgery, there are five instructors, three of whom are required to teach the practical and mechanical branches to thirty pupils; and our calculation is, that for each additional thirty, another operative and mechanical demonstrator must be added, so that for a class as large as that ordinarily in attendance at the larger medical schools, about twenty-four teachers would be required. Moreover, in the dental college the whole of five months has been found barely sufficient for giving instruction in practical and mechanical dentistry to graduates of medical schools. One whole month previous to lectures, is devoted to mechanical and operative dentistry, and during the lecture session, the students devote five or six hours a day to the same exercises. Yet Dr. Gardette thinks that all this work can be done equally well by an adjunct professor, who shall be an attachè to a medical faculty, lecturing when they will give him an opportunity—to students when they will give him a hearing. Does Dr. G. believe that any lecturing, however excellent, would make such operators as he has seen made in a dental college? And does he not know that if the University of Pennsylvania were seriously to set themselves to work to teach their students dentistry, their whole building would not be sufficient to make the necessary operating rooms?

But we feel that these objections are too obvious to need enforcement; it is obvious that Dr. G. does not contemplate that any practical instruction in dentistry should be given in the medical colleges. How this very essential part of a dentist's education is to be got he does not suggest.

That medical faculties may be found who will undertake to teach dentistry is possible. There are few things some of them would not *promise* to do and undertake to do, if students could be lured by the novelty. But that any of them will ever enlarge their operations to such an extent as really to educate their students as dental surgeons, is to us utterly improbable. The project may find favor. Dental lecturers may be appointed by trustees and faculties who are themselves utterly unqualified to discriminate between a dentist and a quack. Students may be lured away from dental colleges—these institutions may be abandoned—mechanical dentistry may descend to the goldsmith's, and then dental surgery will have become a dishonored appendage to medicine. The young physician will regard it as a catch-penny attachment to his legitimate pursuit—to be used to fill up the vacancies of medical practice, to be a biscuit boy to his profession—an alimentary resource upon which he can rely upon a pinch. It would be easy to fill up the details of such a sketch, but our readers can do this for themselves.

In conclusion, we beg leave to remark, that the utter destruction of one profession is too much to pay for a complimentary connexion with medicine. If dentistry is to be elevated, we would show a more excellent way. Instead of multiplying difficulties in the way of dental colleges let all that have love or hope for the profession go zealously to work to build them up. If half the students of dentistry now in the offices of dentists should be sent to the dental colleges, they would be able to take rank, at once, with the best of medical schools. They would increase their professorships, enlarge their buildings, increase their museums, augment their publications, and command for the service of the profession, the best talent of the country.

We have merely to add, that the plan suggested by Dr. Gardette. is not a novel one. About thirty-five years ago, the late Dr. Hayden, of Baltimore, delivered courses of lectures on dental surgery, in the University of Maryland—the experiment, however, was unsuccessful.

As we did not receive Dr. Gardette's article until the two last forms of our Journal were about being made up, we have only had time to prepare a few brief comments upon it. On a future occasion, we may examine the subject more at length.

In giving place to the foregoing remarks, we have been compelled to omit several articles which had been prepared for the present number.

